# A rare case of Fournier's gangrene developing from colon perforation in an infant: A case report

**DOI:** 10.1016/j.eucr.2024.102721

**Published:** 2024-03-29

**Authors:** Fiki Setiawan, Jupiter Sibarani

**Affiliations:** Department of Urology, Faculty of Medicine University of Padjadjaran, Bandung, Indonesia

**Keywords:** Fournier's gangrene, Necrotizing fasciitis, Infection of perineum and genitalia

## Abstract

Fournier's gangrene, a rare and life-threatening soft tissue infection affecting the genitalia and perineum, results from various microorganisms. This rapidly progressing necrotizing fasciitis yields higher mortality and morbidity rates. We report a case of a 1-month-old male infant with Fournier's gangrene due to perforation transverse colon complicated with septic shock and pneumonia also accompanied by patent processus vaginalis. Radiological findings of pneumonia and pneumoperitoneum were exhibited. Early diagnosis and management are demanded to mitigate life-threatening and improve the prognosis. The patient underwent incision drainage, peritoneal lavage, exploratory laparotomy, colostomy, necrotomy debridement, and patent processus vaginalis ligation after hemodynamic status stabilization.

## 1 Introduction

Fournier's gangrene (FG) is an unusual and rarely found infective necrotizing fasciitis of genitalia and perineum causing life-threatening conditions associated with high mortality and morbidity. It is well recognized that this condition is not exclusive to young males, but can affect both genders in all ages. Several studies have reported that the age range changed from infancy to adulthood.^1^^,^^2^ Fournier's gangrene can affect individuals across a wide range of ages, typically occurring in men aged 50 to 79, with an incidence of 0.4 per 100,000 adults. Fournier gangrene can also occur in healthy individuals but is more commonly associated with those who are debilitated or immunocompromised, such as male diabetics over 50 years old with a history of alcohol abuse. Originally described as presenting mainly in healthy young males, the age range for Fournier's gangrene has expanded to include early infancy up to adulthood. There have been reports of this condition occurring in children in the first week of life, highlighting the potential for this disease to manifest at a very young age. Although limited cases have been reported in children, it is essential to recognize that Fournier's gangrene can affect individuals of various age groups. it is essential to recognize that Fournier's gangrene can affect individuals of various age groups. Fournier gangrene is associated with various risk factors, these risk factors include conditions such as diabetes, alcohol abuse, recent trauma or surgery, and comorbidities like hypertension, spinal cord injury, obesity, and immunosuppression. Specific factors such as atherosclerosis, chronic steroid use, colorectal malignancy, HIV infection, inflammatory bowel disease, liver failure, and male gender also increase the likelihood of Fournier gangrene. Additionally, certain medical treatments like chemotherapy and the use of sodium-glucose cotransporter 2 inhibitors in diabetes have been linked to an increased risk.

Necrotizing fasciitis cases of different anatomic localizations, including the perineal, have been reported in comparison of 0.08 per 100.000 in children. Fournier's gangrene in the pediatric population can have various underlying risk factors and etiologies. In children, predisposing factors for Fournier's gangrene may include trauma, insect bites, circumcision, burns, periurethral and anorectal diseases, systemic infections, immunocompromised states, hematologic malignancies, premature, low birth weight, immunocompromised, and poor personal hygiene. The source of infection leading to Fournier's gangrene in children can be urogenital, anorectal, or cutaneous. Manifestation in the early stages is usually not specific and diagnosis is done based on a highly suspected condition.^3^ In the present, we report a 1-month-old infant with Fournier's gangrene.

## 2 Case presentation

**Fig. 1:**
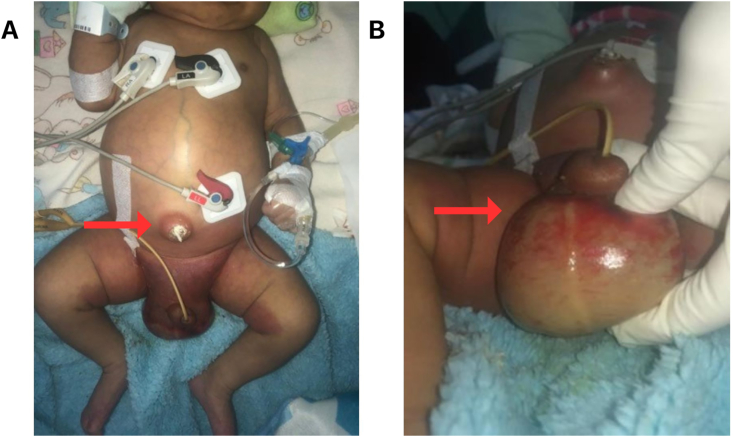
The patients clinically displayed inflammation in the umbilical and scrotal region.

A 1-month-old male infant presented with swelling in the scrotal wall for 5 days. Swelling in the scrotal extended to the lower abdomen and both thighs. Complaint was accompanied with fever and shortness of breath that was getting worse. His parents also complained that the end of the umbilical cord had never dried since birth and had been oozing pus. The patient is the first child, born with the help of a traditional birth attendant. Patient's birth weight was 3800 g. On physical examination the patient was in septic shock condition, revealing scrotal wall swelling, erythema with necrotic tissue, and fluctuation. The umbilical region also showed inflammatory signs and a distended abdomen with decreased bowel sounds (Fig. 1).

Blood investigations showed a white blood count of 8540/mm^3^, platelet count of 10000/mm^3^, serum urea of 136.6 mg/dL, serum creatinine of 1.45 mg/dL, albumin of 1.84, CRP of 21.05, and procalcitonin of 181.15. Blood gas analysis revealed a pH 7.101, pCO2 of 23.5, pO2 of 77.2, HCO3 of 7.4, tCO2 of 8.1, BE -20, and Sat 90.

**Fig. 2:**
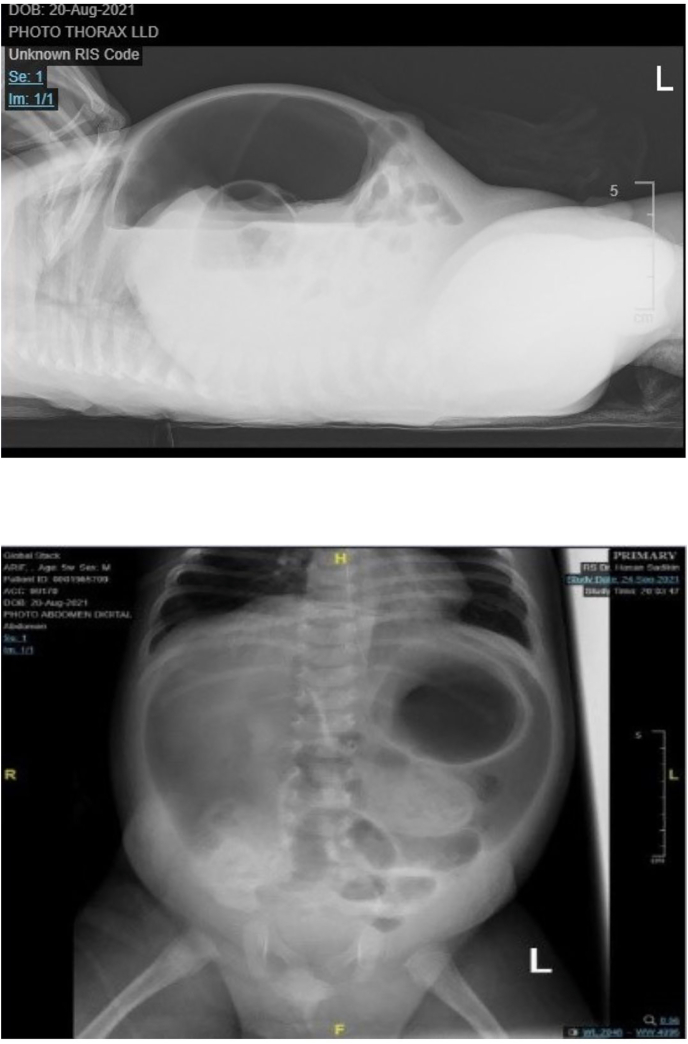
Rontgen abdomen showed pneumoperitoneum.

Chest X-ray displayed pneumonia in the right lung. Pneumoperitoneum also showed in the abdomen X-ray. *Pseudomonas aeruginosa* and *Eschericia coli* showed in the isolated pus culture (Fig. 2).

**Fig. 3:**
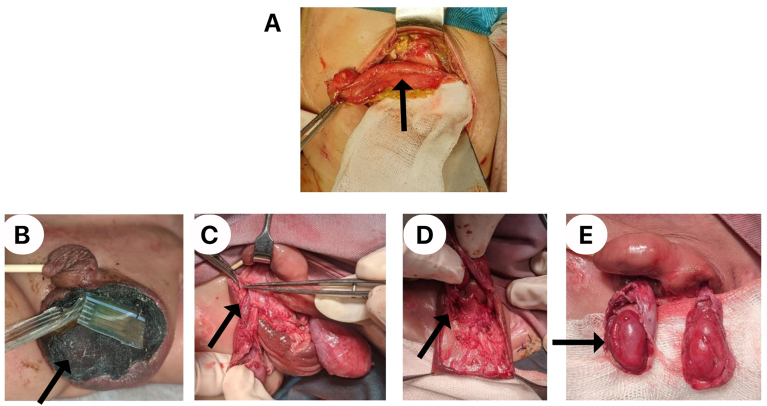
(A) Perforation at transverse colon around 8 cm, (B, C, D, E) Intraoperative finding during necrotomy debridement.

The patient was stabilized by fluid resuscitation, oxygenation, empiric antibiotic, and supportive inotropic. For source control, the patient was treated by incision drainage of the pus in the scrotal wall and performed peritoneal lavage under local anesthesia due to the high risk for anesthesia (Fig. 3). After the hemodynamic was stable and the platelet count was more than 100.000/mm^3^, the patient was performed exploratory laparotomy with high risk for general anesthesia ASA 5E due to the suspected intraperitoneal sepsis.^4^ Intraoperative finding was perforation at transverse colon about 8 cm, then performed transcolectomy and end colostomy proximal transverse. After laparotomy exploration, the patient underwent necrotomy debridement for Fournier's gangrene, and intraoperative patent processus vaginalis was found. After almost one month of hospitalization, patient developed hospital-acquired pneumonia and became a respiratory failure.

## 3 Discussion

Fournier's gangrene (FG) is an aggressive and harmful infection that may quickly progress into severe complications and death in vulnerable patient populations. It may progress as rapidly as 1–2 mm/hr. Mortality rates of FG are in the range of 11%–45%.^2^ In infants younger than 3 months, mortality rates are about 9–30%.^2^ Mostly, Fournier's gangrene is caused by *Streptococcus, Staphylococcus,* and commonly *Pseudomonas aeruginosa*.^3^ The most common source of infection may be urogenital, anorectal, or cutaneous accounting for 45%, 33%, and 21%, respectively.^3^ Infective conditions that increase the risk of FG include perirectal or perianal infections, post-varicella infection, cutaneous HPV lesions, insect bites, diaper rash, testicular torsion, omphalitis, insect bites, and systemic infections.^2^^,^^4^ A case report by Ekingen et al.^2^ reported a 17-month-old female patient who developed a similar case after varicella infection. The present case showed omphalitis for 5 days before admission. The patient born assisted by a traditional birth attendant with poor hygiene might increase the risk of FG. Children with significant edema where tissue integrity is compromised, such as in nephrotic syndrome may be at risk for developing FG due to the compromised tissue integrity and susceptibility to infections associated with this condition. Furthermore, children with nephrotic syndrome may have additional risk factors for FG, such as diabetes and immunocompromise, which can further increase their susceptibility to severe infections like FG.

Characteristics of FG are abrupt onset, rapid progression, and absence of a specific etiologic agent. Early symptoms include swelling, erythematous, and tenderness of the involved area which can mimic other diseases as well as cellulitis, viral illness, erysipelas, etc. Infection initially starts in the deep fascia resulting in prominent pain, high fever, and systemic toxicity. The swelling and crepitus of the scrotum progresses rapidly, and dark purple areas develop resulting in extensive scrotal gangrene.^2^ Clinically, the present case highly indicated Fournier's gangrene with complication of septic shock.

Diagnosis of FG is usually established based on the patient's clinical findings. Additional laboratory tests and histopathologic studies can be helpful in the diagnosis of FG. Blood culture and gram staining of the involved tissue may aid in identifying the etiology and choosing appropriate antibiotics. Even though it's not possible to develop microorganisms in any patient, *Escherichia coli*, *Bacteroides*, *Streptococcus*, *Peptostreptococcus*, and *Clostridium* spp. are frequently identified as causative microorganisms in repeated polymicrobial infection.^2^ The etiology of the colonic perforation in our study is suspected to be a secondary event, descending from the peritoneum due to a perforative transverse colon and the presence of a patent processus vaginalis, suggesting it as a subsequent complication rather than the primary cause. The patient underwent exploratory laparotomy and necrotomy debridement for Fournier's gangrene (FG), during which the colonic perforation was identified and managed accordingly, confirming its secondary occurrence in the disease progression. This case report highlights the colon perforation as a contributing factor to the development of FG in the infant patient, with intraoperative findings revealing a perforation at the transverse colon, approximately 8 cm in size. The presence of a perforated colon can contaminate surrounding tissues with fecal matter, bacteria, and toxins, fostering conditions conducive to necrotizing fasciitis like FG.

A similar complaint of swelling scrotum by a ten-month-old infant was reported by Johannes et al.^1^ Changes in skin color to black and becoming foul smelling were also reported.^1^ However, no complications followed this progressively infective condition. The current case demonstrated a complication of sepsis and pneumoperitoneum on radiologic findings. The isolated culture revealed infection of *Pseudomonas aeruginous* and *Escherichia coli.* A previous report also showed a 14-month-old male patient with perineal necrotizing fasciitis who also grew *Pseudomonas aeruginosa* in the tissue culture.^3^ However, other causative organisms were also implicated, such as *Enterococcus* and *S. aureus*.^3^

The pathogenesis of this disease is explained as an invasion of bacteria into subcutaneous tissue and spreading horizontally through deep fascial planes. Bacterial toxin release leads to ischemia of tissue and necrosis and causes fulminant systemic disease.^3^ Suppurative bacterial infection may cause microthrombosis in small vessels and induce the growth of gangrene in the involved skin.^5^ Sheehy et al.^5^ reported a similar case in a male adult patient. The gangrene was preceded by acute or chronic pancreatitis. The computed tomography scan showed fluid tracking from the pancreas into the retroperitoneum, accessing down to the perineum.^5^ Few cases of FG which developed through local spreading of infection following rectum and sigmoid perforation had been reported in previous literature. In this present case, the infection descended from the peritoneum due to perforative transverse colon and the presence of patent processus vaginalis.

The principles treatment for FG is to stabilize the patient and start the antibiotics as soon as possible. It includes hemodynamic support, fluid resuscitation, surgical debridement, broad-spectrum antibiotics, and supportive care. After the patient is stabilized, the following step is the excision of devitalized tissue aggressively, as the necrosis may progress rapidly in hours. Broad-spectrum antibiotic combinations as antimicrobial treatment should be initiated before surgery, and either changed or continued according to results of tissue culture.^1^^,^^2^ In the present case, the patient was stabilized by fluid resuscitation, given O_2_ 5 lpm via simple mask, and inotropic. Antibiotics also started to be given. Previous case covered the patient empirically with the combination of ceftriaxone, metronidazole, and cloxacillin.^1^

Frequent wound irrigation (every 6–8 hours) for wound care after surgical debridement includes with saline and dressing changes and application of an antibacterial ointment. If the wound is clean and granulation tissue develops, one can either perform reconstructive surgery or allow closure by secondary intention. Allowing these wounds to heal by secondary intention may prolong hospital stay.^2^^,^^3^ In a study by Ameh et al.^4^ mentioned that they had also given tetanus prophylaxis when the patients were not immunized yet or had unknown immunization status to prevent tetanus complications in FG. Untreated FG may rapidly expand to sepsis condition and cause multiple organ failure.^5^

The infant in the present case has been in septic shock condition. The patient also had morbidities, such as bronchopneumonia and peritonitis at the time of admission, proven by the thoracic and abdominal rontgen. The patient in this report was successfully treated by exploratory laparotomy and colostomy, followed by necrotomy debridement and ligation patent processus vaginalis when the condition became stable. After almost one month of hospitalization, the patient fell into respiratory failure due to hospital-acquired pneumonia.

## 4 Conclusion

Fournier gangrene in infants is a rare case with a high mortality rate. A mixture of aerobic and anaerobic microorganisms is usually the underlying etiology. A prompt diagnosis should be made to obtain the underlying cause and the complications that may develop. Therefore, early and appropriate management can be conducted to avoid the life-threatening condition. With a multidisciplinary approach, the patient may have a good outcome.

## Consent

The consent of the patient's family regarding the usage of the patient's medical record has been acquired.

## Formatting of funding sources

This research did not receive any specific grant from funding agencies in the public, commercial, or not-for-profit sectors.

## CRediT authorship contribution statement

**Fiki Setiawan:** Writing – review & editing, Writing – original draft, Resources, Methodology, Investigation, Formal analysis, Conceptualization. **Jupiter Sibarani:** Visualization, Validation, Supervision, Resources, Methodology, Conceptualization.

## Declaration of competing interest

None.

## References

[1] Delport J.E., Makamba K. (2020). Necrotising fasciitis in a ten month old infant. Urol Case Rep.

[2] Ekingen G., Isken T., Agir H., Öncel S., Günlemez A. (2008). Fournier's gangrene in childhood: a report of 3 infant patients. J Pediatr Surg.

[3] Zundel S., Lemaréchal A., Kaiser P., Szavay P. (2017). Diagnosis and treatment of pediatric necrotizing fasciitis: a Systematic review of the Literature. Eur J Pediatr Surg.

[4] Ameh E.A., Dauda M.M., Sabiu L., Mshelbwala P.M., Mbibu H.N., Nmadu P.T. (2004). Fournier's gangrene in neonates and infants. Eur J Pediatr Surg.

[5] Sheehy S.-A., Kelly M.E., Francis E.C., Sweeney K.J., Hussey A. (2016). A rare case of Fournier's Gangrene. J Surg Case Rep.

